# “There Was a Sense That Our Load Had Been Lightened”: Evaluating Outcomes of Virtual Ethics Rounds for Veterinary Team Members

**DOI:** 10.3389/fvets.2022.922049

**Published:** 2022-07-18

**Authors:** Anne Quain, Siobhan Mullan, Michael P. Ward

**Affiliations:** ^1^Faculty of Science, Sydney School of Veterinary Science, University of Sydney, Sydney, NSW, Australia; ^2^University College Dublin, Dublin, Ireland

**Keywords:** ethics rounds, moral case deliberation, clinical ethics support services, moral distress, ethical challenge, veterinary ethics

## Abstract

Clinical ethics support services (CESS) are employed in healthcare to improve patient care and help team members develop skills to recognize and navigate ethically challenging situations (ECS). The objective of this study was to evaluate the impact of ethics rounds, one form of CESS, on veterinary team members. An anonymous, online mixed-methods survey incorporating a 15-item instrument designed to assess the outcomes of moral case deliberation originally developed for human healthcare workers (the Euro-MCD 2.0), was developed. The survey was administered to veterinary team members prior to and following participation in a 90-min virtual ethics rounds session. A total of 23 sessions of virtual ethics rounds were held. In total, 213 individuals participated, and 89 completed both surveys (response rate 41.8%). Most respondents were female (*n* = 70, 81%). Most were veterinarians (*n* = 51, 59%), followed by other veterinary team members (practice manager, animal attendant) (*n* = 18, 21%), veterinary nurses or animal health technicians (*n* = 10, 12%) and veterinary students (*n* = 8, 9%). Age ranged from 20 to 73 (median 41, IQR 32–52, *n* = 87). While there was no statistically significant difference between overall modified Euro-MCD 2.0 scores between T1 and T2, there were statistically significant changes in 7 out of 15 Euro-MCD 2.0 items in the domains of moral competence and moral teamwork. Reflexive thematic analysis of free-text responses identified themes including the types, impact and barriers to resolving ECS, the impacts of ethics rounds on veterinary team members and constraints preventing veterinary team members from speaking up in the face of ECS. While participants largely described the impact of ethics rounds as beneficial (for example, by facilitating clarification of thinking about ECS, allowing participants to see ECS from the perspective of others and providing a safe space for discussion), reflecting on ECS could be stressful for participants. Active participation in ethics rounds may be inhibited in the context of power imbalance, or in settings where bullying occurs. Overall, carefully facilitated ethics rounds has the potential to improve the ability of veterinary team members to identify and navigate ECS, and potentially mitigate moral distress.

## Introduction

Veterinary team members commonly encounter ethically challenging situations (ECS), a potential source of moral distress which may negatively impact wellbeing, in their daily work (1–8). Moral distress is defined as “the experience of psychological distress that results from engaging in, or failing to prevent, decisions or behaviors that transgress, or come to transgress, personally held moral or ethical beliefs” (9). Among healthcare workers, moral distress has been correlated with low psychological empowerment and autonomy, low workplace satisfaction and engagement, poor ethical climate and collaboration, high turnover and career attrition (10–12). Moral distress among healthcare workers is also correlated with reduced quality of care, including reduced patient safety and reduced treatment efficacy (12–14). Where clinicians are distressed and/or inadequately supported, their capacity to provide care in a timely, competent and compassionate manner is diminished (15). Similarly, moral distress among veterinary team members may be associated with job turnover and career attrition (16), and may negatively impact the quality of care provided, thus having a detrimental effect on animal welfare. In a report on veterinary practice team wellbeing, Strand argues that emotional labor and moral distress can cause veterinary team members to have “short fuses” and escalate team conflict, which in turn negatively impacts team morale and patient care (17).

It is argued that the primary goal in addressing moral distress is to address the moral or ethical issues that cause the distress (18). In the healthcare sector, ethics training has been shown to help reduce moral distress (12). In the veterinary sector, understanding and application of ethics is identified as a key day-1 competency by the World Organisation for Animal Health (OIE) (19), and accrediting bodies including the Royal College of Veterinary Surgeons (UK) (20), the European Association of Establishments for Veterinary Education (Europe) (21), and the North American Veterinary Medical Education Consortium (22). Yet veterinarians do not feel that their training adequately equips them to navigate ECS successfully (1, 3, 4). Furthermore, the moral reasoning of practicing veterinarians was found to be no greater than the general public with regard to animal ethics, regardless of years of experience (23). These findings have prompted calls for better training of prospective veterinary team members in ethics (3–5, 23, 24). While a survey of veterinary undergraduate curricula in Europe documented improvements in the teaching of animal welfare science, ethics and law overall in the period from 2013 to 2020, 37% of institutions still only partially met, or did not meet, Day-1 ethics competencies (25), indicating scope for further improvement in undergraduate curricula, and flagging the potential need for opportunities to develop ethics competencies after graduation.

Additionally, Millar argued that “beyond early career training, there is also an increasing need to support the development of a broader set of ethical reflection skills within the veterinary profession that goes beyond just raising awareness and knowledge acquisition” (26). Organizational support is critical in facilitating positive coping strategies (12). Organizational support includes creating a culture of ethical reflection and discussion, stimulating open dialogue among colleagues and with patients, and investing in medical ethics education for staff. Clinical ethics support services (CESS), the provision of formal and informal advice and support on ethical issues arising from clinical practice and patient care, is a key form of organizational support utilized in human healthcare settings since the 1970s (27, 28). Key factors driving the establishment of CESS in human healthcare included advances in intensive and critical care, organ transplantation, and prominent North American court cases concerned around end-of-life decision making and management (29, 30). Similar trends have been documented in the veterinary sector (31–33). Strand recommends that practices “hold a weekly 1-h moral de-stress meeting” to counter the impacts of moral distress and emotional labor (17).

### Types of Clinical Ethics Support

Fournier divides CESS into two broad approaches, both of which ultimately aim to improve patient care and improve awareness of ECS: clinical ethics consultations (CEC) and moral case deliberation (MCD) (34). The former is focused on resolving an ECS associated with a clinical case as it unfolds in real time. In general, CECs are a “top down” approach, involving an individual or committee that provides expert advice or recommendations regarding a specific patient or case (27, 28). The committee is required to have the collective knowledge and skills to ultimately provide effective recommendations (35). This approach has been utilized in large veterinary teaching hospitals managing complex cases. For example, a clinical ethics committee at North Carolina State University Veterinary Hospital was established to provide clinical consultative services, as well as play an advisory role in policy review and development (36). The committee is comprised of four veterinary faculty members, a social worker, three veterinary technicians, and at the time its work was published, was in the process of recruiting one or two community representatives (35). This CEC adopted the CASES approach, which involves clarifying the ethical challenge, assembling appropriate information including data and opinions, synthesizing ethically appropriate actions, explaining recommendations, offering support, and soliciting feedback to improve subsequent deliberations (35). In this model, veterinary team members seeking CEC are given a written summary, detailing “morally acceptable options,” with reference to factors considered (36). Such approaches may reduce moral distress by providing a consensus view or distributing moral responsibility (35). In some cases, CECs are perceived to have formal authority to advise on case management (27). According to Tapper, the ethics consultation was borne out of dual fears of providing futile care, and concerns about physicians having to address increasing complex ECS (and any medicolegal consequences) alone (30). Yet evidence supporting the efficacy of CECs of addressing these concerns is scarce.

Critics of “top down” approaches argue that they may discourage ethical thinking by delegating responsibility to “experts” (27, 37). There is a concern that ethical expertise, when applied by “outsiders” to a unique situation, may overlook key experiences and insights of clinicians and critical contextual factors (38). Furthermore, CECs may not alleviate moral distress if the consultant's recommendation(s) are in conflict with the moral or ethical beliefs of veterinary team members, if they are perceived to be imposed (37), or if they are perceived to be ignored by clinical decision makers (39). The involvement of “experts” may be resisted by veterinary team members “who do not want treatment decisions taken out of their hands” (37). It is possible that veterinary teams may be more likely to accept solutions developed by themselves than an external party (40). Moses, who has offered CEC services in veterinary teaching hospitals, reported difficulties in providing CECs in real time, as few patients were under care “long enough for someone to notice the ethical nature of a conflict, ask for a consultation, and have it done in the time frame during which decisions must be made” (41). Indeed, in a cross-sectional survey of healthcare workers at a large tertiary academic medical centre, barriers to accessing CECs included concerns that it may slow decision making down, lack of awareness of the existence of a CEC, prior experience of a poor quality consultation, or lack of specific guidance from the CEC (39). Moses observed that a key barrier to the use of CECs was a general lack of ethical literacy within the veterinary profession, such that ECS and associated distress were often not identified as ethical in nature (41). If an ECS is not recognized as such, a request for a CEC may not be triggered. Another limitation of CECs is that they require a number of expert members, which may not be feasible in a small workplace (26).

For the above reasons, “bottom-up” approaches, including MCD, ethics rounds, clinical ethics review, ethics discussion groups, and ethics reflection groups (15, 27), may be more helpful in veterinary settings. Rather than relying on the deliberation of experts, bottom-up approaches utilize facilitator-led, structured discussions of one or more ethical challenges specific to a particular setting (42), aiming to support participants in managing ECS (43). These approaches draw on the experiences and insights of the participants (for example, healthcare workers) themselves (38). The facilitator does not have authority (27), but may assist in clarifying the ethical question or source of moral distress, and introduce existing ethical theories or concepts, as well as normative frameworks such as laws, codes and policies that may support or constrain particular decisions (38). The facilitator plays a role in balancing normative and restorative elements, that is, elements of MCD that may restore team member wellbeing such as learning that one is not alone (42). Their role is to help overcome misunderstandings or conflict by highlighting common values, or views, and fostering respect and tolerance about different ethical positions (44). Typically, the topic is chosen by participants (15, 27). Bottom-up approaches enable participants to formulate ethical questions, review facts, norms, values, decision points leading to a particular outcome, opportunities and constraints for decision makers, and alternatives at each decision point, establish common ground between different stakeholders, and gain new insights into an ECS or type of ECS (15, 27, 45). They may help team members to clarify their ethical values, identify and navigate ECS, and may also help mitigate negative impacts of ECS, including moral distress and burnout (27, 43, 44, 46, 47). Participation in these discussions may play a restorative role in helping participants process their thoughts and feelings about ECS they have encountered (27, 42). Through this process, participants may shift from a feeling of moral distress or unresolved moral conflict, toward increased clarity about what might have or should have been done in the circumstances (15).

Sessions typically run for 45–90 min, and involve groups of 5–12 participants (46). The facilitator utilizes specific techniques such as Socratic dialogue or the hermeneutic method to draw participants on their values, or provide language and conceptual tools like ethical frameworks to clarify the moral dimensions of healthcare or veterinary work (15, 34, 44). This approach has been trialed with veterinarians at the Division of Small Animal Internal Medicine at the University of Veterinary medicine in Vienna (40). Additionally, Springer et al. (28) described an Equine Hospital Ethics Working Group, established at the University of Veterinary Medicine in Vienna in 2015, that largely followed the clinical ethics consultation model, but transitioned to a bottom-up approach, as the discussion lead to reflection on general ethical issues. Hobson-West and Millar developed small group facilitated discussions for final year veterinary students to discuss ECS they had encountered (48), consistent with a bottom-up approach.

Potential benefits of bottom-up CESS are described in relation to the patient, the healthcare team and the organization, and outlined in Table 1.

**Table 1:** Potential benefits of bottom-up clinical ethics support services at the level of the patient, healthcare team and organization.

**Level**	**Potential benefit**	**References**
Patient	• Improved patient care •Improved interaction with patients and family members	(27) (49)
Healthcare team	•Increased ethical awareness/ability to identify and articulate ethical challenges •Improved awareness of own behavior and thinking, improved ability to post-pone moral judgements, listen critically and sincerely •Improved skills in navigating ethical conflict •Improved understanding of the perspectives of others •Improved multidisciplinary cooperation •Provision of a safe space for disagreement •Emotional relief and validation A reminder that individuals do not have to navigate complex ECS alone •Insight into moral responsibility, allow team members to feel heard, facilitate acceptance of moral discomfort •Strengthened confidence to act in the face of ECS	(50) (38) (44) (50) (28) (50, 51) (15, 51) (15) (15) (51)
Organization	•Facilitate improved understanding across and between disciplines, underscore the need for some team members to be explicit about their ethical decision making, foster inclusion of all team members in ethical deliberations, allow participants to recognize where ECS emerge due to organizational shortcomings that may be subsequently addressed •Promote good governance, and improve institutional culture •Establishment of “paradigm cases” which This may improve efficiency in navigating ECS, and may prevent escalation of ECS.	(51) (28) (28, 40)

While both top-down and bottom-up CESS are utilized to improve patient care, it is argued that the central focus of CEC is on decisions impacting the patient, while the focus of bottom-up approaches like ethics rounds and MCD is the ethical awareness and competence of the healthcare team (27, 28). Empirical evidence regarding CESS is scarce (52). To the authors' knowledge, the impact of CESS, specifically ethics rounds, on veterinary team members has not been evaluated. We sought to conduct a study to determine whether ethics rounds may be beneficial for veterinary team members.

## Methods

In this study, veterinary team members were asked to complete an initial survey, participate in ethics rounds, and complete a second survey. Below we outline methods for each step in this process.

### Recruitment and Consent

Participants were recruited *via* two means. The first was through veterinary organizations contacted by the researchers, who agreed to act as an intermediary between participants and researchers, suggesting session times and emailing links to the survey to participants. The second was direct, where participants could respond to an advertisement posted on the Sydney School of Veterinary Science facebook and twitter accounts. Participants could be within or outside of Australia. Participants recruited *via* veterinary organizations participated in ethics rounds with colleagues, while those who responded to the advertisement were grouped according to which date was most convenient for them to participate. This grouping was based on convenience. Respondents (individual or organizational) were emailed possible dates and times of sessions. Once they confirmed availability for a certain date and time, a link for the survey was sent either direct to participants or to their organization ~48–72 h prior to the session. Following the session, a link was sent direct to participants or to their organization, for the follow up survey, 48–72 h following the session. A final reminder was sent to participants or their organization 48 h after that. No further emails were sent.

To meet inclusion criteria, respondents were required to be a veterinarian, veterinary team member or veterinary student over the age of 18. Participation was open from September 1 to December 31 2021. The landing page of the survey was a participant information statement providing detailed information about the purpose of the study, estimated time required (2 × 5–7 min to complete online surveys, in addition to the 90-min virtual ethics rounds session), information about data storage, the process for providing feedback or making complaints, and assurance regarding confidentiality and anonymity of responses. Participants were advised that clicking the “submit response” button after completing the surveys indicated consent to participate. The study was approved by the University of Sydney Human Research Ethics Committee (project 2021/550).

### Surveys Before and After Virtual Ethics Rounds

We developed a survey comprising 21 questions (see Supplementary Table 1). Participants who had not taken the survey before were asked general demographic information, including gender, role and age. The survey incorporated the European Moral Case Deliberation Outcomes (Euro-MCD) Instrument 2.0, modified for veterinary team members. The Euro-MCD, a rigorously-developed, 26-item instrument, was originally developed in 2014 to measure outcomes of moral case deliberation across six domains (enhanced emotional support, enhanced collaboration, improved moral reflexivity, improved moral attitude, improvement on organizational level and concrete results) (43). The Euro-MCD was developed in a multi-national context, and has been utilized in a variety of healthcare settings. The Euro-MCD 2.0, developed in 2020, consists of 15 items across three domains [moral competence (items 1–6), moral teamwork (items 7–11), and moral action (items 12–15)] (43). Minor adjustments were made to the items. Notably, as there is variation between veterinary team members regarding whether something is experienced as an ethical challenge or not (3), we replaced the term “ethically difficult situations” with “ethically challenging situations.” Additionally, as veterinary teams refer to animals as patients and owners or guardians as clients, we replaced the word “families” in the last two items with “clients.” Participants who had taken the survey before and participated in ethics rounds were asked whether they had anything to add about ECS they encountered in their work, and whether they had anything they wished to add about ethics rounds.

We utilized the Research Electronic Data Capture (RedCAP) survey platform, a secure web application hosted by the University of Sydney, to build and manage the survey. The survey was piloted by clinical veterinarians (four) and veterinary academics (three). All feedback that improved clarity of the questions was incorporated into the final survey. To ensure that surveys prior to and after participant in virtual ethics rounds could be compared for each respondent, after consultation with the Sydney University Research Data Consultant, respondents were asked to enter a unique code consisting of their mother's initials, year of birth of their mother, and their father's initials. These were concatenated in RedCAP. On responding to the second survey, respondents were asked to enter this same information. This facilitated pairing of responses. Once responses were paired by matching these details, each participant was given a participant number (1–89), and the unique identifier was removed. Data were stored on the physically and electronically secure, restricted-access University of Sydney server, which is routinely backed up and accessible only to the authors.

### Virtual Ethics Rounds

The term “ethics rounds” has previously been utilized in the veterinary literature, albeit briefly and infrequently, and largely limited to educational settings, to describe group ethical discussions. In 1983, Graber described “ethics grand rounds” involving a case review, identification of ethical value issues associated with the case, and discussion of the central ethical issue culminating in a decision explicitly justified by a group leader, or a group voting on a decision (51). At this time, participants were encouraged to seek the “right” answer. The authors provide 24 case studies with leading questions, suggesting a “top down” approach to what constitutes an ECS. Erde and Pollock described an elective ethics summer school, arguing that it would be “optimal” for the teaching of veterinary ethics throughout the degree, such that “these periodic discussions would culminate in the fourth year in which one afternoon of each rotation would be devoted to “ethical rounds” and in which a resident ethicist would participate in hospital grand rounds and other case discussions” (52). The focus in these approaches is on helping veterinary students make difficult decisions, but no mention is made of moral distress, nor is there explicit emphasis on students nominating ECS to discuss. We utilized the term “ethics rounds” rather than “moral case deliberation,” as we found that veterinary team members we engaged with were more likely to understand the former as designating a reflective, multi-disciplinary group discussion, and most were unfamiliar with the latter. Thus for the purposes of this discussion, “ethics rounds” is used interchangeably with “moral case deliberation.”

The structure of ethics rounds was adapted from small-group facilitated sessions for final year veterinary students at the University of Nottingham, described by Hobson-West and Millar (48). A schedule of the session is outlined in Supplementary Table 2. While we had initially anticipated holding sessions in person in both Australia and the United Kingdom, the imposition of movement restrictions and physical distancing due to the COVID-19 pandemic prohibited such gatherings, with no clear endpoint for such restrictions. Furthermore, travel outside of Australia for the purposes of research was not permitted. Thus the decision was made to host rounds virtually, utilizing Zoom (Zoom Video Communications, Inc), a cloud based video-conferencing platform. All sessions were facilitated by the first author (AQ), a female veterinarian with 16 years experience in veterinary clinical practice, and 10 years experience in teaching at academic institutions. In addition to veterinary and higher education qualifications, the facilitator had completed a Bachelor of Arts degree majoring in philosophy, co-authored a textbook of veterinary ethics (53) and run numerous workshops on managing ECS in veterinary clinical contexts, with veterinary students and clinicians. She is trained in psychological first-aid.

The first part of the session involved the facilitator introducing the concepts of moral stress, moral distress and moral injury in relation to ECS, followed by a brief discussion of the potential risks and benefits of ethics rounds. The rules of the session were outlined as follows: the content of the session was confidential, though the facilitator/researcher would make handwritten notes of general themes discussed, and write down key observations about running of the session; participation was voluntary and participants could leave at any time, and participants were encouraged to leave seniority and rank behind and avoid assigning blame. Participants were also informed that no video or audio recordings of the sessions would be made, in order to insure their privacy.

After the introduction of rules, participants were asked to suggest any additional parameters or rules around the discussion. For the purposes of discussion, an ECS was defined as “a situation where we are required to manage competing choices, or where there may be conflict between the interests of different stakeholders or parties who may be impacted by a decision.”

The second part consisted of a general discussion of the types of ECS that participants had encountered. Participants could either state these out loud, or write them named or into the Zoom chat, either to all participants, or directly to the facilitator to remain anonymous to other participants. One of these was selected for discussion by a vote or consensus of the participants, after which a 5-min comfort break was provided. Participants were asked to mute their microphone and turn off the camera during the break. The third part consisted of discussion of at least two courses of action, in light of relevant laws or codes of practice, professional responsibilities and key ethical theories. Participants were asked to select and justify a course of action. In the fourth part, the facilitator provided a brief overview of the types of ECS described in contemporary veterinary ethics literature, and encouraged participants to reflect on their learnings from the session, and how they may manage ECS going forward. The facilitator made a final request for participant's comments or questions. At the close of the session, participants were reminded to contact their Employee Assistance Program (EAP), or one of several listed counseling hotlines or webchat resources designed for veterinary professionals (for example, their professional association's counseling service) if they experienced distress. They were reminded to complete the survey following ethics rounds. The timing of each part of the session was variable, to ensure that the facilitator could respond to the flow of the discussion.

### Quantitative Data

Survey data were downloaded from RedCAP onto Micosoft Excel^®^ for Microsoft 365 MSO Version 2112 (Build 14729.20254). Responses were organized according to the unique identifier code. Only those responses with a matching response code were included in the final analysis. If a respondent had completed the survey more than twice, for example twice before (T1) and once after participating in ethics rounds (T2), the most complete or earliest response was retained, with the less complete or later response excluded from analysis. For each respondent, the total Euro-MCD score was calculated for both T1 and T2, in addition to the change in the modified Euro-MCD 2.0 score (T2-T1, “Euro-MCD change score”). Worksheets were imported into IBM^®^ Statistical Package for Social Sciences [SPSS^®^ Statistics Version 26 (Release 26.0.0.0)].

Descriptive analyses were performed by assessing the distribution of categorical variables with frequency tables. The single continuous variable, age, was described using summary statistics. Contingency tables were used to describe the association between categorical variables and the binary outcome variable “increased MCD score vs. not increased MCD score.” The distribution of continuous outcome variables by each category of the outcome variable was described with summary statistics and boxplots. Univariable binary logistic regression analyses were performed to investigate the association of explanatory variables with the outcome variable. For each of the 15 statements on the Euro-MCD, paired *t*-tests were performed to calculate the mean difference (T2-T1) for each item. A *P*-value of ≤ 0.05 was considered statistically significant.

### Qualitative Data

Responses were screened to exclude identifying information prior to being uploaded onto NVivo12 Plus Software (QSR International) to facilitate thematic analysis. For the types of ECS discussed in sessions, as recorded by the facilitator, a codebook analysis was utilized. We used codes developed in a previous thematic analysis of ECS depicted in published vignettes (54). Each ECS was coded once, and coding frequencies recorded. For free-text responses to survey questions, responses were uploaded into separate files to facilitate reflexive thematic analysis of responses to each question. According to best practice, reflexive thematic analysis, as an interpretive activity, should explicitly recognize the researcher's role in the construction of themes (55, 56). The first author's background is described in 2.3. The second author is the Chair in Animal Welfare and Veterinary Ethics at University College, Dublin. She initially worked in mixed then companion animal practice before transitioning from clinical work to focus on research and teaching in the area of animal welfare science, ethics and law. The third author is the Chair of Veterinary Public Health and Food Safety in the Sydney School of Veterinary Science. His veterinary clinical experience is derived exclusively from government practice as a field veterinarian.

Reflexive thematic analysis involved six stages. First, the first author read all comments three times. Second, initial codes were generated. Each comment was coded inductively for semantic themes, employing a realist approach without a pre-existing theoretical framework. An iterative approach was used. Each comment could be coded multiple times. Where a response, or part of a response, could not be assigned to an existing code, a new code was generated. Third, initial themes were generated by identifying clusters of codes, which were grouped together as themes to best represent the data. As part of this stage, themes were reviewed for internal coherence and distinctiveness from other themes. If responses or partial responses did not fit a theme, these were reallocated to a more suitable theme, or to a new theme. The fourth and fifth stages—refining themes, developing thematic maps, and naming themes—were performed concurrently. The sixth and final stage involved selecting illustrative examples for each theme.

## Results

### Quantitative Data

A total of 23 sessions of virtual ethics rounds were run between 14 September and 12 December, 2021. In total, 213 individuals participated in virtual ethics rounds. Group sizes ranged from 2 to 50, with a mean of 9.3 and median of 5.0 (standard deviation 10.7). When outliers were removed, the mean group size was 5.2 with a median of 4 (standard deviation 2.1). The source of participants is described in Table 2. In total, 147 veterinary team members completed the first survey, and 95 completed the second survey. Of these, paired responses were identified for 89 respondents. Therefore, paired surveys from a total of 89 respondents were analyzed, representing a response rate of 41.8% (*n* = 89/213).

**Table 2:** Frequency table describing the number of sources of participants in virtual ethics rounds sessions for veterinary team members (*n* = 23 sessions).

**Source of participants**	**Number of sessions**	Percentage (%)*
Participants from different organizations/workplaces	7	30.4
Animal shelter/animal welfare organization	5	21.7
Government/regulatory veterinary bodies	4	17.4
Veterinary school	4	17.4
Corporate veterinary practice (companion animal)	2	8.7
Private veterinary practice (companion animal)	1	4.3
Total	23	99.9

^*^Column percentages may not add to 100 due to rounding.

The distribution of categorical demographic variables is described in Table 3. The majority of participants were female (*n* = 70, 81%), and most were veterinarians (*n* = 51, 59%), followed by other veterinary team members (practice manager, animal attendant) (*n* = 18, 21%), veterinary nurses or animal health technicians (*n* = 10, 12%), and veterinary students (*n* = 8, 9%). Age in years ranged from 20 to 73, with a median of 41 and an interquartile range of 32–52 (*n* = 87).

**Table 3:** Frequency table for the demographic information on respondents to surveys both prior to and following participation in virtual ethics rounds (*n* = 89).

**Demographic parameter**	**Category**	**Number**	**Percentage (%)**
Gender (*n* = 87)	Female	70	80.5
	Male	17	19.5
	Total	87	100
Role (*n* = 87)	Veterinarian	51	58.6
	Veterinary nurse or animal health technician	10	11.5
	Other e.g., practice manager, animal attendant	18	20.7
	Veterinary student	8	9.2
	Total	87	100

The distribution of responses to items from the modified Euro-MCD instrument 2.0 for respondents prior to the participating in ethics rounds, and following participation in ethics rounds, are described in Table 4. Summary statistics for the outcome Euro-MCD change score overall and by categories of the categorical predictor variables are described in Table 5. There was a significant (*P* < 0.0001) low negative Pearson correlation co-efficient (*r* = −0.14) between age and the Euro-MCD change score. There was a significant (*P* < 0.0001) moderate negative Pearson correlation co-efficient (*r* = −0.63) between the Euro-MCD score at time one, and the Euro-MCD change score overall. All univariable residuals were checked and distribution was approximately normal. All univariable regression analyses were not significant at *P* ≤ 0.05 (see Supplementary Table 3), suggesting no statistically significant change between total scores at T2 when compared with T1.

**Table 4:** Frequency table for responses to statements adapted from the Euro-MCD instrument (2.0) from respondents prior to (T1) and following (T2) participation in virtual ethics rounds (*n* = 89).

**Statements adapted from the Euro-MCD instrument 2.0 (50)**	**Time**	Strongly agree number (%*)	Slightly agree number (%*)	Slightly disagree number (%*)	Strongly disagree number (%*)	Don't know/not applicable number (%*)
I recognize a situation as being ethically challenging	T1	57 (64)	29 (33)	1 (1)	0 (0)	2 (2)
	T2	83 (93)	6 (7)	0 (0)	0 (0)	0 (0)
I am aware of others' perspectives in ethically challenging situations	T1	47 (53)	40 (45)	2 (2)	0 (0)	0 (0)
	T2	56 (63)	33 (37)	0 (0)	0 (0)	0 (0)
I can identify the different values at stake in ethically challenging	T1	36 (40)	44 (49)	7 (8)	1 (1)	1 (1)
situations	T2	65 (73)	23 (26)	1 (1)	0 (0)	0 (0)
I can formulate arguments in favor of and against different courses	T1	37 (42)	44 (49)	6 (7)	0 (0)	2 (2)
of action in ethically challenging situations	T2	58 (65)	31 (35)	0 (0)	0 (0)	0 (0)
I listen with an open mind to others when discussing an ethically	T1	34 (38)	47 (53)	6 (7)	(0)	2 (2)
challenging situation	T2	58 (65)	30 (34)	1 (1)	0 (0)	0 (0)
I speak up in ethically challenging situations	T1	33 (37)	35 (39)	19 (21)	1 (1)	1 (1)
	T2	39 (44)	41 (46)	9 (10)	0 (0)	0 (0)
We openly express our viewpoints in ethically challenging	T1	18 (20)	44 (49)	22 (25)	4 (5)	1 (1)
situations	T2	31 (35)	48 (54)	10 (11)	0 (0)	0 (0)
We all have opportunities to express our viewpoints when	T1	15 (17)	41 (46)	26 (29)	4 (5)	3 (3)
discussing ethically challenging situations	T2	31 (35)	40 (45)	14 (16)	2 (2)	2 (2)
We respect different viewpoints when discussing ethically	T1	20 (23)	46 (52)	15 (17)	4 (5)	4 (5)
challenging situations	T2	49 (55)	31 (35)	8 (9)	0 (0)	1 (1)
We feel secure to share emotions in ethically challenging situations	T1	14 (16)	32 (36)	28 (32)	11 (12)	4 (5)
	T2	28 (32)	48 (54)	8 (9)	3 (3)	2 (2)
We support each other when dealing with ethically challenging	T1	27 (30)	52 (58)	7 (8)	2 (2)	1 (1)
situations	T2	47 (53)	33 (37)	6 (7)	1 (1)	2 (2)
We made decisions on how to act in ethically challenging	T1	18 (20)	51 (57)	7 (8)	5 (6)	8 (9)
situations	T2	40 (45)	38 (43)	10 (11)	0 (0)	1 (1)
We base our decisions on moral considerations in ethically	T1	32 (36)	32 (36)	19 (21)	2 (2)	4 (5)
challenging situations	T2	49 (55)	30 (34)	6 (7)	0 (0)	4 (5)
We are responsive to the values and needs of patients and clients	T1	34 (38)	42 (47)	8 (9)	0 (0)	5 (6)
in ethically challenging situations	T2	49 (55)	35 (39)	2 (2)	1 (1)	2 (2)
We are able to explain and justify our care toward patients and	T1	42 (47)	33 (37)	7 (8)	0 (0)	7 (8)
clients	T2	57 (64)	26 (29)	2 (2)	0 (0)	4 (5)

^*^Row percentages may not add to 100 due to rounding.

**Table 5:** Summary statistics for the outcome Euro-MCD change score overall and by categories of the categorical predictor variables.

**Predictor**	**Minimum**	**25th percentile**	**Mean**	**Standard deviation**	**Median**	**75th percentile**	**Maximum**
**Gender**
Female	−7	1	6	7	5	10	26
Male	−6	3	6	7	5	9	18
**Role**
Veterinarian	−7	0	5	7	4	9	23
Veterinary nurse or animal health technician	−4	0	6	8	5	8	26
Other e.g., practice manager, animal attendant	0	2	7	6	6	10	21
Veterinary student	4	4	8	7	10	13	18
Total	−7	1.5	5.8	6.6	5.0	10.5	26

There were statistically significant changes in 7 out of 15 Euro-MCD 2.0 items, specifically “I recognize a situation as being ethically challenging”; “I can identify the different values at stake in challenging situations”; “I can formulate arguments in favor of and against different courses of action in ethically challenging situations”; “I listen with an open mind to others when discussing an ethically challenging situation”; “we openly express our viewpoints in ethically challenging situations”; “we all have opportunities to express our viewpoints when discussing ethically challenging situations”; and “we respect different viewpoints when discussing ethically challenging situations” (see Table 6). Of the domains, there were statistically significant changes in 4/6 items in the domain of moral competence, 3/5 items in the domain of moral teamwork and 0/3 in the domain of moral action. In the domain of moral competence, there was no statistically significant change in the subdomain of supportive relationships (items 10 and 11).

**Table 6:** Mean difference between item-specific scores on Euro-MCD 2.0 from before and after participation in virtual ethics rounds (*n* = 89).

**Statements adapted from the Euro-MCD instrument 2.0 (50)**	**Mean difference (T2-T1)**	**95% confidence interval**	***P*-value**
I recognize a situation as being ethically challenging	0.6	0.1–1.1	0.013
I am aware of others' perspectives in ethically challenging situations	0.3	−0.1–0.8	0.137
I can identify the different values at stake in ethically challenging situations	0.7	0.2–1.1	0.005
I can formulate arguments in favor of and against different courses of action in ethically challenging situations	0.6	0.1–1.1	0.013
I listen with an open mind to others when discussing an ethically challenging situation	0.6	0.2–1.1	0.010
I speak up in ethically challenging situations	0.5	−0.0–0.9	0.055
We openly express our viewpoints in ethically challenging situations	0.6	0.1–1.1	0.011
We all have opportunities to express our viewpoints when discussing ethically challenging situations	0.6	0.1–1.1	0.013
We respect different viewpoints when discussing ethically challenging situations	0.8	0.3–1.3	0.001
We feel secure to share emotions in ethically challenging situations	2.8	−1.6–7.3	0.203
We support each other when dealing with ethically challenging situations	2.4	−2.0–6.9	0.274
We made decisions on how to act in ethically challenging situations	2.8	−1.6–7.2	0.214
We base our decisions on moral considerations in ethically challenging situations	2.6	−1.8–7.0	0.245
We are responsive to the values and needs of patients and clients in ethically challenging situations	2.5	−1.9–6.9	0.257
We are able to explain and justify our care toward patients and clients	2.5	−1.9–7.0	0.255

### Qualitative Data

In total, there were 143 types of ECS recorded by the facilitator during the sessions. These were coded into 25 out of 29 existing categories. Examples of types of ECS in each category, together with coding frequencies, are included in Supplementary Table 4. The most common ECS fell into the categories of how to manage a client who refuses a recommendation or does not adhere to advice; euthanasia of companion animals; clients with limited finances; and collegial relations and wellbeing of veterinary team members.

In total, there were 48 responses to the question “Is there anything you wish to add about ECS you have encountered in the course of your work?” comprising 1,896 words. We identified eight key themes: types of ECS encountered by veterinary team members, ECS impact veterinary team members, there are barriers to resolving ECS, veterinary team members have a variable degree of autonomy of in making ethical decisions, underlying factors may increase the risk of encountering ECS, there is a need for ethics training for veterinary team members, there are factors that help veterinary team members navigate ECS, and concerns about the survey or terminology used (for examples, see Table 7; for thematic map, see Supplementary Figure 1).

**Table 7:** Themes constructed through reflexive thematic analysis of free-text responses to the question “Is there anything you wish to add about ethically challenging situations you have encountered in the course of your work?” in a survey of veterinary team members following participation in virtual ethics rounds (*n* = 89).

**Theme**	**Example(s)**
Types of ethically challenging situations encountered by veterinary team members	“The usual dichotomy of finances and the need to make money.” “The conflict between animal welfare and human welfare is also a significant challenge.” “…in a professional life, personal morals and ethics have to co-exist alongside regulation. For example, just because I don't like “x”, if it is regulated and permitted for it may happen. perhaps a role of the official veterinary service in this scenario is to be the champion of rigorous adherence to regulation and to keep an open mind to the possibility of improvements and changes in standards and ensure that they lobby for these to be included in the regulations”
Ethically challenging situations impact veterinary team members	“Some situations and events weigh on my mind post-event.” “The personal emotional effect that these situations present can be exhausting.”
There are barriers to resolving ethically challenging situations	“…we often believe that our fundamental beliefs are the right ones and everyone else is somehow not as legitimate a viewpoint as our own.” “I sometimes find it challenging knowing that there will be compromise in either animal needs, owner needs or my professional needs when dealing with ethically challenging situations.” “In the past power has tended to dictate which view wins which is both frustrating and demoralising.” “It's difficult because in some positions it is considered inappropriate to speak up in an ethically challenging situation.” “The “we” as a team does not always include the practice owners. Their viewpoints can be clouded with financial considerations.”
Veterinary team members have a variable degree of autonomy of in making ethical decisions	“Discussion of ethical scenarios within a practice is appropriate. However, if colleagues each have a solid moral compass, then each has the right to decide how to respond to ethical situations which arise.” “As a government employee, at times, I feel that I am not in a position always to question and or deal with ethically challenging situations which are already known to senior personnel.”
Underlying factors that may increase the risk of encountering ethically challenging situations	“Animals are still regarded as chattels despite the closer attachment to the family compared with previous years and also finances play an important part in the decision making for the owners.” “I actually think the profession itself is highly conflicted and has inadequately thought through animal welfare, business interests etc.”
There is a need for ethics training for veterinary team members	“I think we have opinions but may not be skilled to discuss it from ethical points of view, or be aware of how to describe our underlying ethical opinion.” “We are not trained in ethics at uni” “The vet I worked for was very old school so he had a bit of a black and white concept of ethics and didn't really train his workers in this concept. He was less compassionate to those who had to follow through with his instructions.”
There are factors that help veterinary team members navigate ethically challenging situations	“Legislative changes in this area have helped support people who would have refused on ethical grounds.” “We need to recognise how we are viewing the situation and what framework we are using to assess the situation.” “Each situation has to be handled as its own entity, having different context and considerations that need to go into the decision making process.”
Concerns about the survey or terminology used	“Ethically challenging maybe a bit ambiguous as one who feels they have a strong ethical compass may find most situations not at all challenging.” “I found the questions above that referred to “we” [in the MCD instrument] difficult to answer. It's difficult to generalise in a meaningful way about how ethically challenging situations are handled with colleagues due to the wide variety of ethically challenging situations and which colleagues or combinations of colleagues might be involved in dealing with them.”

In total, there were 44 responses to the question “Is there anything you wish to add about ethics rounds?” comprising 1,615 words. We identified five key themes: the benefits of ethics rounds, ethics rounds can have potentially negative impacts on participants, there are constraints preventing veterinary team members from speaking up in the face of ECS, ethics rounds could be improved, and limitations of the Euro-MCD as it pertained to the experience of participants (for examples, see Table 8) (for the thematic map, see Supplementary Figure 2). The benefits of ethics rounds comprised six subthemes: (1) ethics rounds helps clarify thinking, (2) ethics rounds allows participants to see ethical challenges from the point of view of others, (3) ethics rounds provided a safe, supportive forum, (4) ethics rounds can help veterinary team members identify and deal with moral distress, (5) it was validating to discuss ECS, and (6) ethics rounds increased confidence to speak up in the face of ECS.

**Table 8:** Themes constructed through reflexive thematic analysis of free-text responses to the question “Is there anything you wish to add about ethics rounds?” in a survey of veterinary team members following participation in virtual ethics rounds (*n* = 89).

**Theme**	**Subtheme**	**Example(s)**
Benefits of ethics rounds	Ethics rounds helps clarify thinking	“While I probably thought like this, it was helpful to formally break down a ethically challenging situation with respect to stakeholders—their impact on the situation, the impact of the situation on them.” “I found that learning about the different frameworks for thinking about ethical challenges useful for ordering my thoughts and talking about tis with clients/colleagues. Like all the bits and pieces were there before but now I can articulate them better.”
	Ethics rounds allows participants to see ethical challenges from the point of view of others	“In particular I can see a real benefit of it to allow people to discuss ethically challenging situations with work colleagues...irrespective of rank. I think an opportunity to air concerns in an open and frank manner is invaluable for each others state of mind. Even if no specific “answer” is arrived at, it is soothing to know that other colleagues have similar concerns and we can learn from each other's strategies to cope.” “This really helped me understand different viewpoints and how to address them.”
	Ethics rounds provided a safe, supportive forum	“Free and open sharing of ethical issues encountered was facilitated by an excellent facilitator, and colleagues were supportive of one-another.” “They provide a safe space for unpacking and engaging ethically challenging situations.”
	Ethics rounds can help veterinary team members identify and deal with moral distress	“It is such an important area to be aware of. I think many vets and nurses experience moral injury without knowing that is what it is as this is a topic most of us have never heard of. For me personally it has been an absolute revelation that a concept like moral injury exists and it has helped me explain my reactions in so many situations across my career but also privately. I think this has huge potential for helping many vets and associated staff.” “There was a sense that our load had been lightened.”
	It was validating to discuss ethically challenging situations	“Surprisingly helpful in validating team member's stress and concern about the ethical decisions they have to make.”
	Ethics rounds increased confidence to speak up in the face of ethically challenging situations	“Discussing topics with peers was extremely rewarding and made me more confident to speak up in the workplace.”
Ethics rounds can have potentially negative impacts on participants		“While I found the overall experience to be positive, reliving some distressing situations which I had encountered caused me some upset. Distressing situations which I encountered in practice changed the course of my career at different points, and so the impact of those challenging situations was significant.”
There are constraints preventing veterinary team members from speaking up in the face of ethically challenging situations		“Whilst it is pleasant to consider all colleagues working harmoniously, there are differences in opinions which should be respected, but any bullying behavior impacts significantly on one's confidence in self-expression. “Gaslighting” continues to be an industry problem.” “There is a strong level of unspoken intimidation in most clinics where I have worked. The more forceful (usually male) voices dominate and are disparaging toward other, less strong, more timid voices, often subduing these into silence, leaving them longing for the security of darkness and anonymity. There is a far greater issue at stake than just the question of ethics here. As with all things, it appears to be about power.” “I think in future perhaps just team members and no managers should participate. I felt that the team were scared to truly voice some opinions with the managers there.”
Ethics rounds could be improved		“More discussion of what could be done in each of the ethically difficult situations.” “…it was more like a webinar than a rounds session, we talked about ethical situations in general terms but without any specifics which made it hard to come to any conclusions on how we might be able to do things differently in future.” “I think if ethics rounds were more frequent and timely (in relation to a particular event), on-going stress and distress might be less of an issue.”
Limitations of the Euro-MCD as it pertained to the experience of participants		“The challenge in this survey is that there are other considerations not included here, which have an impact upon the decision-making.” “Regarding the comment above about support... I am not sure we know enough about support as a community to support each other with ethically challenging situations. We can mentor, and share opinions... but I'm not sure thats the same as support.”

### General Observations

There was marked variation in group size, due largely to last minute withdrawal of participants, some of whom were required to attend to patients, and the inclusion of four large groups of veterinary students from one institution (comprising groups of 18, 25, 21, and 50, respectively). Not all participants had stable internet connections, which caused minor glitches (not being able to see/hear participants clearly at all times, and occasionally participants not being able to see/hear the facilitator). Aside from the rules suggested by the facilitator, none of the groups suggested any additional rules, nor were any rules objected to. Participants expected the facilitator to have detailed clinical knowledge and be able to point them to resources, notably published data or legislation, which may assist in decision making. Some participants emailed the facilitator following the session seeking publications relevant to ECS they had encountered. It was notable that a number of non-veterinarians felt compelled to clarify that they were not a veterinarian during the discussion. Participants struggled most with identifying alternative courses of action.

## Discussion

To the authors' knowledge, this is the first study seeking to measure the impact of a type of CESS on veterinary team members. Our response rate of 41.8% was good, given response rates to online surveys reported in the order of 25–30% (57). It falls within reported response rates to surveys incorporating the Euro-MCD 1.0, from 23 to 85% (46). The demographic of respondents was similar to that of a previous global survey we conducted of veterinary team members, the majority of whom were female (80.4%), veterinarians (78.3%), with a mean age of 40 (8). The gender balance reflects an overall greater proportion of females in the veterinary workforce (58–62).

While there were no statistically significant changes in the overall Euro-MCD 2.0 score before and after participation in ethics rounds, participants only had a single opportunity to participate in ethics rounds. Additionally, whilst overall there was no significant difference, the relationship is masked by those with higher baseline scores (T1) whose scores were similar at T2. Those with lower baseline scores had more to gain, which is not unexpected. It is possible that those with little experience or training in managing ECS have most to gain from participation in ethics rounds. Our survey did not specifically ask respondents about their previous training or experience, but this can be addressed in future studies.

Ideally, ethics rounds would be held at regular intervals. Clinical ethics support involves both implicit and explicit values (44), which may take time to become apparent. It may take participants several sessions before they are comfortable with the facilitator, utilizing ethical frameworks, or indeed identifying ECS as such. All sessions were held virtually to facilitate social distancing, however it is possible that face-to-face sessions may have facilitated better communication and further enhanced outcomes.

Participants recorded statistically significant improvements in the domains of moral competence and moral teamwork, suggesting that ethics rounds is a promising tool to improve the ethical skills of veterinary team members. Further studies are required to determine if such changes are sustained over time. Interestingly, there was no statistically significant change in any items in the domain of moral action. Additionally, in the domain of moral competence, there was no statistically significant change in the subdomain of supportive relationships. It may be that participants needed to attend more than one session of virtual ethics rounds before impact on moral action was seen. Or it is possible that we surveyed participants too soon after ethics rounds. A longer gap between the intervention and the second survey may have enabled respondents to have encountered more ECS and thus enact ethical decisions. Alternatively, the results may indicate that virtual ethics rounds may not be as effective across certain domains. For example, it may be that in addition to ethics rounds, organizational changes are required to support moral action. Such changes are likely to take time to implement.

A comprehensive discussion of the types of ECS identified by participants is beyond the scope of this paper, however we note that these were consistent with ECS identified in the veterinary ethics literature (1–5, 7, 32, 54). Consistent with published literature, respondents confirmed that ECS had a negative and sometimes long-lasting impact on them (63), that there are numerous barriers to resolving ECS (64), and that there are some factors—such as the legal status of animals as property in most jurisdictions—which increase the risk of encountering ECS (65). Respondents highlighted the need for more training of veterinary team members in identifying and resolving ECS. Surveys of veterinarians and veterinary team members have highlighted concerns about lack of training in navigating ECS, and associated skills such as conflict management (1, 3, 4). According to both individual item modified Euro-MCD 2.0 scores, and thematic analysis, ethics rounds helped veterinary team members identify and approach ECS. Further studies are required to determine if ethics rounds helps veterinary team members to resolve ECS in alignment with their values.

Analysis of free-text comments suggests that organizational changes may be required to ensure veterinary team members feel free to fully engage with ethics rounds. Of concern, some respondents spontaneously reported “bullying,” “intimidation” and feeling “scared.” Bullying behavior has been documented in veterinary workplaces. In a survey of New Zealand veterinarians (*n* = 197), bullying was reported by 16.2% of respondents (66). Mean scores were significantly higher for female compared to male respondents, and non-managers compared to managers. Perceived organizational support moderated the relationship between workplace bullying and strain if bullying scores were low, but had no impact when bullying scores were high. It is possible that the supportive environment of ethics rounds is therefore not sufficient to overcome high levels of bullying. Similarly, veterinary team members may be less likely to engage with ethics rounds in “toxic” veterinary workplaces. According to focus group discussions among Canadian veterinarians (*n* = 23) and registered veterinary technicians (*n* = 26), “toxicity” may manifest as team members being disrespectful, resistant to change, seeking to avoid conflict, lacking in motivation, and experiencing broken communication and tension between staff members (67).

Even where bullying and toxicity are not issues, veterinary team members may be inhibited from speaking up by workplace hierarchies. There may also be perceived or real conflicts between the priorities of employers/managers and employees. In healthcare settings, it has been recognized that the presence of managers may stifle discussion, particularly where the discussion is critical of organizational factors. However, it may be useful for managers to be present, as it can help promote open communication across professional boundaries, and promote mutual respect and understanding. It may also be critical for effecting change at an organizational level. One possible solution is to include managers in a proportion of the meetings, as has been reported in healthcare settings (46).

As noted previously, clinical ethics review or ethics rounds is not an inevitably benign intervention (15, 18). While relief may stem from clarifying the source of emotions that accompany ECS, including frustration, anger, shame and guilt (44), recalling events that gave rise to these emotions may intensify moral distress. While we did not measure moral distress, it was noted that discussing ECS could be distressing. To avoid unintended harms, it is recommended that facilitators have the specific skillset to create a psychologically safe environment for discussion, and explain the nature, scope, safe application and limits of ethics rounds to participants before and after proceeding (15). In some cases, other forms of support, including psychological first aid, counseling or critical-incident stress debriefing may be more suitable (15, 42). While it has been recommended that practices hold regular meetings to discuss situations that lead to moral distress (17), we would encourage the engagement of a facilitator with an appropriate skill set to minimize risks to participants. Delany et al. (15) recommend that critical-incident stress debriefing be provided 1–2 weeks after a challenging event, to allow those involved time to process emotional aspects of the event. While there is currently no evidence to support or challenge such a guideline regarding ethics rounds, given the emotional salience of ECS, a precautionary approach might be to limit discussion of ECS discussed in ethics rounds to those that have occurred 1–2 weeks ago.

Thematic analysis revealed that participants experienced many benefits associated with participating in ethics rounds, as have been noted in published literature. Clarification of thinking about ECS, seeing ECS from the perspective of others, and providing a safe space to discuss ECS are all important steps in helping veterinary team members resolve ECS that they may encounter. Overall, ethics rounds as a form of CESS is a tool that has the potential to equip veterinary team members with the skills to identify and navigate ECS, and potentially mitigate moral distress.

### Limitations

Participation in this study was voluntary. Research based on subject self-selection is particularly prone to sample bias. Additionally, the voluntary nature of the surveys may have increased non-response bias, leading to underrepresentation of some cohorts and over-representation of others (68).

As noted previously, virtual delivery may have inhibited discussion. However, given the ongoing COVID-19 pandemic and the potential for future pandemics, it is possible that many workplace meetings will continue to be held virtually. The virtual format minimized logistic considerations such as finding a suitable venue, facilitated participation of veterinary team members from different and sometimes distant locations, and minimized financial and environmental costs associated with requiring participants to attend in-person.

In this study, there was marked variation in group size, in part due to a high drop out rate and also due to the inclusion of four groups of students. Group size may impact the dynamic and thus the experience of ethics rounds. We concur with Silen et al. (46) that group size should be capped at 12.

Surveys were anonymous to maximize participant privacy, of critical importance given that the researcher was also the facilitator of all sessions. However, this prevented clarification of responses. The surveys were only available in English, and the facilitator does not fluently speak languages other than English, limiting participation to participants who can speak fluent English. This study design did not allow us to compare outcomes between participants from different countries, as cultural and contextual factors including geographic location can impact the types of ECS encountered, and associated moral distress. Future studies may facilitate comparison of results between respondents from different countries. As mentioned previously, in evaluating the impact of ethics rounds it may be useful in future studies to incorporate questions about the types, quantity and quality of previous ethics training that participants had been exposed to.

The study design does not permit follow-up to determine if changes in Euro-MCD 2.0 scores are sustained over time. Additionally, participants in this study only attended a single session of ethics rounds. Ideally, ethics rounds are held on a regular basis (46). The results suggest that more sessions are needed to reliably measure meaningful change due to ethics rounds.

This study relies on self-assessment, which may be subject to social desirability bias. Socially desirable responding is characterized by providing answers that align with social norms, rather than truthful answers, and can result in underestimation of the prevalence of socially undesirable attributes, and overestimation of the prevalence of socially desirable attributes (69, 70). The Euro-MCD does not measure outcomes relating to sick leave, employee turnover or patient outcomes (43). We did not measure levels of moral distress in participants, and so we are not able to determine how, if at all, participation impacted moral distress. There is limited published research exploring the relationships between moral distress, modifying factors, psychological wellbeing, job satisfaction and career attrition in veterinary team members (63). There are a number of potential instruments that have been developed and utilized to measure moral distress in healthcare workers (71–74). These may be useful to explore in future studies of veterinary team members.

## Data Availability Statement

The datasets generated for this article are not readily available because we have approval to disseminate aggregated data, but not individual data. Requests to access the datasets should be directed to anne.quain@sydney.edu.au.

## Ethics Statement

The studies involving human participants were reviewed and approved by Human Research and Ethics Committee, University of Sydney. The patients/participants provided their written informed consent to participate in this study.

## Author Contributions

AQ: literature review, study design, survey building and piloting, ethics application, ethics rounds coordination and correspondence, ethics rounds facilitation, data analysis, writing, editing, and submission. SM: study design, survey refinement, ethics application, ethics rounds coordination, data analysis, editing, and supervision. MW: study design, survey refinement, ethics application, data analysis, editing, and supervision. All authors contributed to the article and approved the submitted version.

## Funding

The open access publication fee is supported by the Sydney School of Veterinary Science.

## Conflict of Interest

The authors declare that the research was conducted in the absence of any commercial or financial relationships that could be construed as a potential conflict of interest.

## Publisher's Note

All claims expressed in this article are solely those of the authors and do not necessarily represent those of their affiliated organizations, or those of the publisher, the editors and the reviewers. Any product that may be evaluated in this article, or claim that may be made by its manufacturer, is not guaranteed or endorsed by the publisher.
