# Supplementary material for: “There Was a Sense That Our Load Had Been Lightened”: Evaluating Outcomes of Virtual Ethics Rounds for Veterinary Team Members
Source: Front Vet Sci. 2022 Jul 18;9:922049. doi: 10.3389/fvets.2022.922049 (PMC9339959; doi:10.3389/fvets.2022.922049)
Supplement: Supplementary file 1 [file Data_Sheet_1.docx]

Supplementary Material

# Supplementary tables

**Table 1.** Ethics rounds session schedule adapted from [1].

| **Section** | **Approximate time allocation** | **Content** |
| --- | --- | --- |
| Part 1 | 10 minutes | Facilitator introduction  Introduction to concepts of moral stress/distress/injury, and ethics rounds as an intervention utilised in human healthcare, including potential risks and benefits of ethics rounds.  Ground rules   - Confidentiality - Impartiality - Blameless - Dynamic (participants free to change their position) |
| Part 2 | 15 minutes | Briefly describe a specific or type of ethically challenging situation you have witnessed.  Select an example to work through |
|  | 5 minutes | Comfort break |
| Part 3 | 20 minutes | Identify at least two courses of action available.  Justify each by reference to:   1. Any relevant laws/codes of practice 2. Your professional responsibilities; 3. Key ethical theories (utilitarianism, deontology, principalism, virtue ethics, Fraser’s practical ethic)   Facilitator helps with a-c. |
|  | 10 minutes | Decide on which course of action is the most justified and why. This may or may not be what was done at the time. |
| Part 4 | 20 minutes | Reflect on what has been learned from this example   1. What are your feelings now about the ethically challenging situation? 2. Are there wider implications? 3. Have you learnt anything about yourself or others through the actual event or discussion today? |
|  | 5 minutes | How might you manage ethical challenges in light of today’s discussion? |
|  | 5 minutes | Close – request for further comments or questions; reminder of confidentiality; review of support resources. |

**Supplementary Table 2.** Survey of veterinary team members pre- and post- participation in ethics rounds.

| **Please rate the extent to which you agree to the following statements, when thinking about**  **your daily practice. If you have participated in ethics rounds, answer these questions in light**  **of the session you have participated in. Note "we" refers to the people with whom you work**  **(i.e. colleagues).** | | | | | |
| --- | --- | --- | --- | --- | --- |
| Statements adapted from the Euro-MCD instrument 2.0 [2] | Strongly agree | Slightly agree | Slightly disagree | Strongly disagree | Don’t know/Not applicable |
| Moral competence  *Subdomain: moral sensitivity* | | | | | |
| I recognise a situation as being ethically challenging | ⃝ | ⃝ | ⃝ | ⃝ | ⃝ |
| I am aware of others perspectives in ethically challenging situations | ⃝ | ⃝ | ⃝ | ⃝ | ⃝ |
| *Subdomain: Analytical Skills* | | | | | |
| I can identify the different values at stake in ethically challenging situations | ⃝ | ⃝ | ⃝ | ⃝ | ⃝ |
| I can formulate arguments in favour of and against different courses of action in ethically challenging situations | ⃝ | ⃝ | ⃝ | ⃝ | ⃝ |
| *Subdomain: Virtuous attitude* | | | | | |
| I listen with an open mind to others when discussing an ethically challenging situation | ⃝ | ⃝ | ⃝ | ⃝ | ⃝ |
| I speak up in ethically challenging situations | ⃝ | ⃝ | ⃝ | ⃝ | ⃝ |
| Moral teamwork  *Subdomain: Open dialogue* | | | | | |
| We openly express our viewpoints in ethically challenging situations | ⃝ | ⃝ | ⃝ | ⃝ | ⃝ |
| We all have opportunities to express our viewpoints when discussing ethically challenging situations | ⃝ | ⃝ | ⃝ | ⃝ | ⃝ |
| We respect different viewpoints when discussing ethically challenging situations | ⃝ | ⃝ | ⃝ | ⃝ | ⃝ |
| *Subdomain: Supportive relationships* | | | | | |
| We feel secure to share emotions in ethically challenging situations | ⃝ | ⃝ | ⃝ | ⃝ | ⃝ |
| We support each other when dealing with ethically challenging situations | ⃝ | ⃝ | ⃝ | ⃝ | ⃝ |
| Moral action  *Subdomain: moral decision-making* | | | | | |
| We made decisions on how to act in ethically challenging situations | ⃝ | ⃝ | ⃝ | ⃝ | ⃝ |
| We base our decisions on moral considerations in ethically challenging situations | ⃝ | ⃝ | ⃝ | ⃝ | ⃝ |
| *Subdomain: responsible care* | | | | | |
| We are responsive to the values and needs of patients and clients in ethically challenging situations | ⃝ | ⃝ | ⃝ | ⃝ | ⃝ |
| We are able to explain and justify our care towards patients and clients | ⃝ | ⃝ | ⃝ | ⃝ | ⃝ |
| The following questions were only asked if the respondent indicated that they had already participated in ethics rounds. | | | | | |
| Gender?  Drop down menu listing the following:   - Female - Male - Other | | | | | |
| Please choose one option that best describes your role (Drop down menu listing the following):   - Veterinarian - Veterinary nurse or animal health technician - Other (if other, please specify) | | | | | |
| What is your age in years? (please enter a whole number) __ | | | | | |
| Is there anything you wish to add about ethics rounds? (Please be careful not to include potential  identifying information such as the names of individuals or workplaces in your response).  (free text response) | | | | | |
| Is there anything you wish to add about ethically challenging situations you have encountered in the course of your work? (Please be careful not to include potential identifying information such as the names of individuals or workplaces in your response).  (free text response) | | | | | |

**Supplementary Table 3.** Univariable linear regression analysis for Euro-MCD change score (for supplementary)

| Predictor | Coefficient (B) | Standard error | 95% confidence interval for B lower | 95% confidence interval for B upper | P-value | R-squared |
| --- | --- | --- | --- | --- | --- | --- |
| (Constant) | 5.9 | 2.3 | 1.4 | 10.5 | 0.01 | 0.00 |
| Gender | -0.2 | 1.8 | -3.9 | 3.4 | 0.91 | 0.00 |
| (Constant) | 3.9 | 1.4 | 1.1 | 6.7 | 0.01 | 0.03 |
| Role | 1.0 | 0.7 | -0.3 | 2.3 | 0.14 | 0.03 |
| (Constant) | 8.5 | 2.3 | 3.9 | 13.2 | <.001 | 0. 02 |
| Age | -.07 | 0.05 | -0.17 | 0.04 | 0.21 | 0.02 |

**Supplementary Table 4.** Types of ethically challenging situations raised by virtual ethics rounds participants, categorized using codebook analysis, in descending order of frequency, with examples.

| **Theme** | **Example(s)** | **Frequency** |
| --- | --- | --- |
| How to manage a client who refuses a recommendation or does not adhere to advice | Client refusal to euthanase an animal with poor/deteriorating welfare or pursue recommended treatment. | 15 |
| Euthanasia of companion animals | Client requests euthanasia for reasons that veterinary team member does not agree with/does not believe are in the interests of the animal. | 14 |
| Clients with limited finances | Clients elect euthanasia on economic grounds.  Clients cannot afford recommended diagnostics or treatment. | 14 |
| Collegial relations and wellbeing of veterinary team members | How to manage conflict between veterinary team members, for example regarding case management.  Pandemic specific challenges – should vaccination of veterinary team members be mandated? How to balance health and safety of the veterinary team with animal welfare/client interests. | 14 |
| Management of stray or unowned dogs and cats | How to assess animals appropriately when their behaviour may be negatively impacted by fear (particularly cats).  How to distribute limited resources (staff time, financial investment, shelter) to stray or unowned animals in shelters. | 10 |
| What should veterinary team members do when clients breach welfare laws or regulations? | Under what circumstances should veterinary team members report clients for beaching animal welfare laws or regulations (for example, clients who own a banned breed or exotic species, clients who engage in animal hoarding)?  How should veterinary team members respond to requests from clients to breach legislation/codes of practice? (e.g., requests to alter records to increase the chances of the client receiving an insurance pay out). | 10 |
| Working with or assisting other team members who are providing incompetent care | How to manage when another team member is not performing to standard, utilizing out of date techniques (e.g., inadequate analgesia).  Working with senior team members/employers who are abusive, violent or cruel to humans or animals. | 7 |
| Shared decision making and informed consent | How to manage if the client is not available to provide consent to an urgently required intervention.  Managing when multiple parties have an interest in an animal or animals and may not all consent.  What to do when a colleague does not appear to obtain consent before proceeding with diagnostic or treatment plan. | 7 |
| Animal welfare (AW) governance | To what extent should animal welfare legislation and regulations be strictly enforced?  What parameters should veterinary registration bodies take into account when investigating concerns that impact animal welfare?  How can complaint investigation bodies ensure that individuals are not penalised when complaints arise due to systemic issues? (e.g., some registration bodies are limited to only being able to investigate the individual named in a complaint, and will hold them responsible, even if the practice culture is the problem). | 7 |
| What forms of animal use are acceptable? | Is it acceptable for veterinarians to support practices associated with poor welfare (e.g., live export of unweaned calves, non-stun slaughter)?  Is it acceptable to facilitate animal use where its impacts on the environment are negative?  Is it acceptable to treat “pest” species? | 6 |
| Futile or non-beneficial treatment of animal patients | Where do you draw the line on non-beneficial treatment?  What constitutes over-treatment?  Should some procedures be disallowed altogether in some species (e.g., wing amputation in birds) | 6 |
| Conflict between the interests of animals and the interests of their owners | How do you deal with concerns about animal welfare when the owner(s) have mental health issues?  How do you manage situations where an owner’s lifestyle prevents them from meeting the needs of their animals? | 5 |
| Slaughter and killing of farm animals | Is culling of animals in a disease outbreak situation ethically justifiable?  Is non-stun slaughter for religious reasons ethically justifiable?  When should farm animals be treated vs slaughtered?  What methods of stunning/euthanasia should be used? | 5 |
| Breeding animals and selecting for particular traits | How do veterinary team members provide care for breeds with conformation that negatively impacts welfare (e.g., brachycephalic obstructive airway syndrome in brachycephalic breeds), without promoting such breeds?  Is it acceptable for veterinary team members to breed animals, especially where those breeds are known to have conformation which negatively impacts welfare?  How should veterinary team members manage relationships with breeders to ensure they are not complicit in poor welfare? | 5 |
| Management of errors and complications | What should a veterinary team member do if they discover a colleague has made an error, but has not admitted this to the client? What if the colleague actively covered up the error? | 4 |
| Scope of practice | How should veterinary team members balance the need to gain experience with their responsibility of practicing within their scope of experience?  When is it acceptable to “have a go” at something you have not tried before? | 3 |
| Treatment and management of wild and free roaming animals | How should decisions be made around whether to treat or euthanase wildlife?  How should resources for wildlife care be distributed? | 3 |
| Conflict of interest (COI) | How should veterinary team members manage conflicts of interest? (e.g., volunteer ethics committee members are needed to make up a quorum but may have conflicts of interest). | 1 |
| How to balance animal productivity with animal welfare | Is it acceptable to keep an injured/unwell animal alive to maximise costs that can be salvaged by the farmer? (e.g., keeping a gravid, valuable breeding animal alive until she has given birth). | 1 |
| Labelling and use of pharmaceuticals including antimicrobials | To what extent should veterinary drug use be impacted by concerns about human health? (e.g., knowing that a particular veterinary agent used to alleviate suffering in animals has been used in suicide, should the profession lobby to restrict access to that agent?). | 1 |
| Standard of care (SOC) | Should veterinary team members perform and offer only “gold standard” care, or should they offer a spectrum of care? | 1 |
| Convenience surgeries and mutilations | Should veterinary team members perform or facilitate convenience surgeries and mutilations where these are widespread and expected (e.g., tail docking). | 1 |
| Competition between veterinarians and practices | How should you manage conflicts of interests created by competition between practices? (e.g., you are repeatedly asked for second opinions on the work of a local competing practice). | 1 |
| Remuneration and charging for veterinary services and product sales | Is it acceptable to charge high fees for advanced veterinary treatment which does not have a good outcome? | 1 |
| Assessment and measurement of animal welfare and quality of life | How do you determine humane end points? (e.g., for experimental animals with a disease that reduces their quality of life) | 1 |

**Supplementary Table 5.** Themes constructed through reflexive thematic analysis of free-text responses to the question “Is there anything you wish to add about ethics rounds?” in a survey of veterinary team members following participation in virtual ethics rounds (n=89).

| **Theme** | **Subtheme** | **Example(s)** |
| --- | --- | --- |
| Benefits of ethics rounds | Ethics rounds helps clarify thinking | “While I probably thought like this, it was helpful to formally break down a ethically challenging situation with respect to stakeholders - their impact on the situation, the impact of the situation on them.”  “I found that learning about the different frameworks for thinking about ethical challenges useful for ordering my thoughts and talking about tis with clients/colleagues. Like all the bits and pieces were there before but now I can articulate them better.” |
|  | Ethics rounds allows participants to see ethical challenges from the point of view of others | “In particular I can see a real benefit of it to allow people to discuss ethically challenging situations with work colleagues...irrespective of rank. I think an opportunity to air concerns in an open and frank manner is invaluable for each others state of mind. Even if no specific 'answer' is arrived at , it is soothing to know that other colleagues have similar concerns and we can learn from each other’s strategies to cope.”  “This really helped me understand different viewpoints and how to address them.” |
|  | Ethics rounds provided a safe, supportive forum | “Free and open sharing of ethical issues encountered was facilitated by an excellent facilitator, and colleagues were supportive of one-another.”  “They provide a safe space for unpacking and engaging ethically challenging situations.” |
|  | Ethics rounds can help veterinary team members identify and deal with moral distress | “It is such an important area to be aware of. I think many vets and nurses experience moral injury without knowing that is what it is as this is a topic most of us have never heard of. For me personally it has been an absolute revelation that a concept like moral injury exists and it has helped me explain my reactions in so many situations across my career but also privately. I think this has huge potential for helping many vets and associated staff.”  “There was a sense that our load had been lightened.” |
|  | It was validating to discuss ethically challenging situations | “surprisingly helpful in validating team member's stress and concern about the ethical decisions they have to make.” |
|  | Ethics rounds increased confidence to speak up in the face of ethically challenging situations | “Discussing topics with peers was extremely rewarding and made me more confident to speak up in the workplace.” |
| Ethics rounds could be improved |  | “More discussion of what could be done in each of the ethically difficult situations.”  “…it was more like a webinar than a rounds session, we talked about ethical situations in general terms but without any specifics which made it hard to come to any conclusions on how we might be able to do things differently in future.”  “I think if ethics rounds were more frequent and timely ( in relation to a particular event), on-going stress and distress might be less of an issue.” |
| There are constraints preventing veterinary team members from speaking up in the face of ethically challenging situations |  | “Whilst it is pleasant to consider all colleagues working harmoniously, there are differences in opinions which should be respected, but any bullying behaviour impacts significantly on one's confidence in self-expression. 'Gaslighting' continues to be an industry problem.”  “There is a strong level of unspoken intimidation in most clinics where I have worked. The more forceful (usually male) voices dominate and are disparaging towards other, less strong, more timid voices, often subduing these into silence, leaving them longing for the security of darkness and anonymity. There is a far greater issue at stake than just the question of ethics here. As with all things, it appears to be about power.”  “I think in future perhaps just team members and no managers should participate. I felt that the team were scared to truly voice some opinions with the managers there.” |
| Ethics rounds can have potentially negative impacts on participants |  | “While I found the overall experience to be positive, reliving some distressing situations which I had encountered caused me some upset. Distressing situations which I encountered in practice changed the course of my career at different points, and so the impact of those challenging situations was significant.” |
| Limitations of the Euro-MCD as it pertained to the experience of participants |  | “The challenge in this survey is that there are other considerations not included here, which have an impact upon the decision-making.”  “Regarding the comment above about support... I am not sure we know enough about support as a community to support each other with ethically challenging situations. We can mentor, and share opinions... but I'm not sure thats the same as support.” |

**Supplementary Table 6.** Themes constructed through reflexive thematic analysis of free-text responses to the question “Is there anything you wish to add about ethically challenging situations you have encountered in the course of your work?” in a survey of veterinary team members following participation in virtual ethics rounds (n=89).

| **Theme** | **Example(s)** |
| --- | --- |
| Types of ethically challenging situations encountered by veterinary team members | “The usual dichotomy of finances and the need to make money”.  “The conflict between animal welfare and human welfare is also a significant challenge.”  “…in a professional life, personal morals and ethics have to co-exist alongside regulation. For example, just because I don't like 'x' , if it is regulated and permitted for it may happen. perhaps a role of the official veterinary service in this scenario is to be the champion of rigorous adherence to regulation and to keep an open mind to the possibility of improvements and changes in standards and ensure that they lobby for these to be included in the regulations” |
| There are barriers to resolving ethically challenging situations | “…we often believe that our fundamental beliefs are the right ones and everyone else is somehow not as legitimate a viewpoint as our own.”  “I sometimes find it challenging knowing that there will be compromise in either animal needs, owner needs or my professional needs when dealing with ethically challenging situations.”  “In the past power has tended to dictate which view wins which is both frustrating and demoralising.”  “It's difficult because in some positions it is considered inappropriate to speak up in an ethically challenging situation.”  “The 'we' as a team does not always include the practice owners. Their viewpoints can be clouded with financial considerations.” |
| Veterinary team members have a variable degree of autonomy of in making ethical decisions | “Discussion of ethical scenarios within a practice is appropriate. However if colleagues each have a solid moral compass, then each has the right to decide how to respond to ethical situations which arise.”  “As a government employee, at times, I feel that I am not in a position always to question and or deal with ethically challenging situations which are already known to senior personnel.” |
| There are factors that help veterinary team members navigate ethically challenging situations | “Legislative changes in this area have helped support people who would have refused on ethical grounds.”  “We need to recognise how we are viewing the situation and what framework we are using to assess the situation.”  “Each situation has to be handled as its own entity, having different context and considerations that need to go into the decision making process.” |
| Underlying factors that may increase the risk of encountering ethically challenging situations | “Animals are still regarded as chattels despite the closer attachment to the family compared with previous years and also finances play an important part in the decision making for the owners.”  “I actually think the profession itself is highly conflicted and has inadequately thought through animal welfare, business interests etc.” |
| There is a need for ethics training for veterinary team members | “I think we have opinions but may not be skilled to discuss it from ethical points of view, or be aware of how to describe our underlying ethical opinion.”  “We are not trained in ethics at uni”  “The vet I worked for was very old school so he had a bit of a black and white concept of ethics and didn't really train his workers in this concept. He was less compassionate to those who had to follow through with his instructions.” |
| Ethically challenging situations impact veterinary team members | “Some situations and events weigh on my mind post event.”  “The personal emotional effect that these situations present can be exhausting.” |
| Concerns about the survey or terminology used | “Ethically challenging maybe a bit ambiguous as one who feels they have a strong ethical compass may find most situations not at all challenging.”  “I found the questions above that referred to 'we' [in the MCD instrument] difficult to answer. It's difficult to generalise in a meaningful way about how ethically challenging situations are handled with colleagues due to the wide variety of ethically challenging situations and which colleagues or combinations of colleagues might be involved in dealing with them.” |

**Supplementary Figure 1.** Thematic map of themes constructed through reflexive thematic analysis of free-text responses to the question “Is there anything you wish to add about ethically challenging situations you have encountered in the course of your work?” in a survey of veterinary team members following participation in virtual ethics rounds (n=89).

**
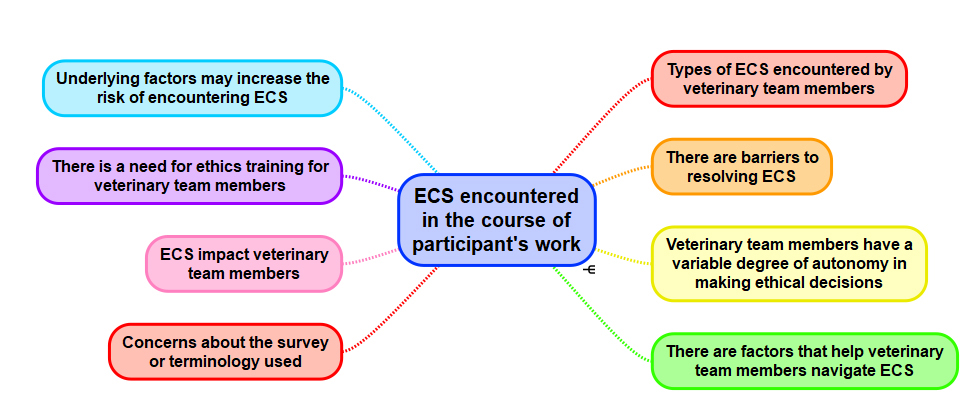
**

**Supplementary Figure 2.** Thematic map of themes constructed through reflexive thematic analysis of free-text responses to the question “Is there anything you wish to add about ethics rounds?” in a survey of veterinary team members following participation in virtual ethics rounds (n=89).

**
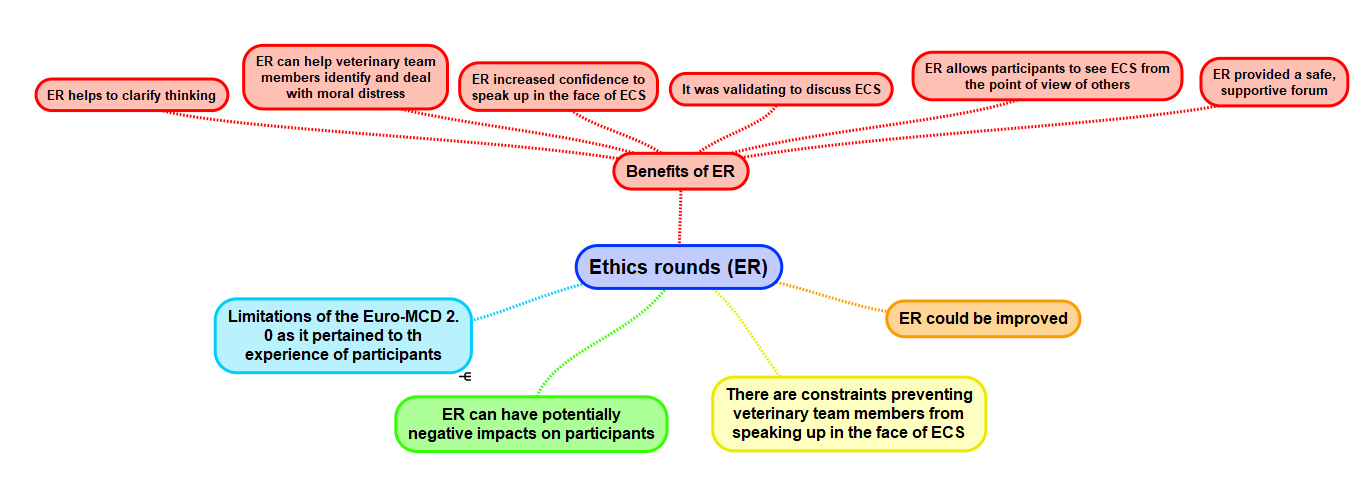
**

**Reference**

1. Hobson-West, P. and K. Millar, *Telling their own stories: Encouraging veterinary students to ethically reflect.* Veterinary Record, 2021. **n/a**(n/a).

2. de Snoo-Trimp, J.C., et al., *Moral competence, moral teamwork and moral action - the European Moral Case Deliberation Outcomes (Euro-MCD) Instrument 2.0 and its revision process.* BMC Medical Ethics, 2020. **21**(1): p. 53.
